# Beyond the INR: Thromboelastography Carries Prognostic Information in Critically Ill Patients With Cirrhosis

**DOI:** 10.1155/cjgh/5532035

**Published:** 2026-07-20

**Authors:** Franklyn K. Wallace, Puru Rattan, Ryan J. Lennon, Alice Gallo De Moraes, Douglas A. Simonetto

**Affiliations:** ^1^ Department of Internal Medicine, Mayo Clinic, Rochester, Minnesota, USA, mayo.edu; ^2^ Division of Gastroenterology and Hepatology, Mayo Clinic, Rochester, Minnesota, USA, mayo.edu; ^3^ Department of Quantitative Health Sciences, Mayo Clinic, Rochester, Minnesota, USA, mayo.edu; ^4^ Division of Pulmonary and Critical Care Medicine, Mayo Clinic, Rochester, Minnesota, USA, mayo.edu

## Abstract

**Background:**

In patients with cirrhosis, prolonged international normalized ratio (INR) is primarily driven by liver synthetic dysfunction. Thromboelastography more accurately reflects coagulability and can also predict short‐term mortality. Nevertheless, INR is used to define coagulation failure in the European Foundation for the Study of Chronic Liver Failure criteria for acute‐on‐chronic liver failure (ACLF).

**Aim:**

To evaluate the association between TEG parameters and mortality in critically ill patients with cirrhosis and to justify future investigation into TEG as a prognostic tool in ACLF.

**Methods:**

We performed a retrospective study of 52 patients with cirrhosis admitted to the intensive care unit (ICU) who had TEG performed during their ICU admission prior to the administration of any blood products. We assessed the association between TEG parameters and 28‐day mortality.

**Results:**

Patients who did not survive beyond 28 days generally had more hypocoagulable TEG parameters (R time: 10.0 vs. 7.6, *p* = 0.03; K time: 3.4 vs. 2.2, *p* = 0.003; alpha angle: 53.1 vs. 63.5, *p* = 0.002; MA: 49.2 vs. 60.2, *p* = 0.001). Although global assessment of TEG demonstrated a state of rebalanced hemostasis among critically ill patients with cirrhosis, patients meeting diagnostic criteria for ACLF tended to have more hypocoagulable TEG parameters as compared to those who did not (coagulation index: 0.2 vs. 2.0, *p* = 0.004). As proof of concept, MA was incorporated into the definition of ACLF to replace INR as a marker of coagulation failure (TEG‐ACLF). The area under the curve (AUC) for TEG‐ACLF was 0.83 (0.72–0.95) compared to 0.8 (0.68–0.92) for the current ACLF‐CLIF grading, with 9.6% (5/52) of patients being reclassified into a different ACLF grade.

**Conclusion:**

Hypocoagulable TEG parameters are associated with increased 28‐day mortality in critically ill patients with cirrhosis, and incorporation of TEG parameters into a modified definition for ACLF may result in improved prediction of short‐term mortality.

## 1. Introduction

Coagulability in patients with cirrhosis is governed by a principle of “rebalanced hemostasis” wherein procoagulant and anticoagulant factors synthesized by the liver are equally deficient, resulting in a tenuous hemostatic balance which is particularly susceptible to the influence of endothelium‐derived coagulation factors in times of stress [1]. It is well‐established that traditional laboratory tests such as the international normalized ratio (INR) and platelet count do not accurately estimate the risk of major bleeding or thrombosis in patients with cirrhosis [2–5]. Thromboelastography (TEG) is a functional assessment of clotting performed on samples of whole blood, which appears to provide a more accurate global assessment of clotting function in patients with advanced liver disease [6–8].

In patients with liver disease, the most heavily investigated clinical application for TEG has been for guidance of blood product administration. As compared to traditional laboratory assays, it has been repeatedly demonstrated that TEG‐guided transfusions in patients with cirrhosis result in less blood product use without increasing major bleeding events [9–14]. TEG‐guided transfusion protocols are increasingly implemented in clinical practice, particularly in hospitalized patients awaiting liver transplantation [15–18]. Indeed, the most recent SCCM guidelines for the Management of Adult Acute and Acute‐on‐Chronic Liver Failure (ACLF) suggest using TEG over traditional markers of coagulation for guiding blood product administration [19].

Less explored, however, is a growing body of evidence supporting the prognostic value of TEG in patients with cirrhosis. There have been several recent publications which have demonstrated an association between hypocoagulable TEG parameters and severity of liver disease (as assessed by traditional methods such as MELD score) in both the outpatient and inpatient setting [20, 21]. Of particular interest is a prospectively designed study investigating hospitalized patients with decompensated cirrhosis, which suggested that TEG parameters, especially clotting time (R time), predicted short‐term mortality even more accurately than the INR [22]. Despite this, the CLIF–Consortium definition for ACLF uses an INR > 2.5 as the defining feature for coagulation failure [23]. Given that the INR is more strongly related to liver function than to a true representation of coagulation failure, and liver function is represented elsewhere in the ACLF definition (bilirubin > 12), the current framework for identifying patients with ACLF may contain unnecessary redundancy, which could be improved upon [23].

For this study, our primary aim was to assess the prognostic value of TEG in the prediction of 28‐day mortality among critically ill patients with cirrhosis. Secondarily, we sought to investigate the role of integrating TEG parameters into the current definition of ACLF.

## 2. Methods

### 2.1. Study Design, Outcomes, and Analysis

**FIGURE 1:**
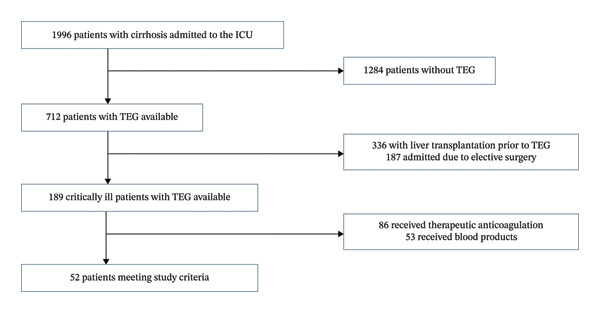
Patient selection.

A retrospective study was performed involving patients with cirrhosis admitted to the intensive care unit (ICU) at Mayo Clinic from 2017 to 2020. The study protocol was approved by the Mayo Clinic Institutional Review Board. All methods were performed in accordance with the relevant guidelines and regulations. Only patients with established research authorization were included. Patient data were retrieved from a previously‐described prospectively‐collected ICU database [24, 25]. The presence of cirrhosis was determined by either (1) biopsy or (2) clinical evidence of gastroesophageal varices, decompensation, or radiologic features of cirrhosis and portal hypertension (i.e., nodular liver contour, evidence of portosystemic collaterals, and splenomegaly) in a patient with chronic liver disease based on diagnostic codes in the electronic health record (EHR) [24]. Patients who had TEG performed during their ICU admission were identified using site‐specific laboratory codes. Through manual EHR review, remaining patients were excluded based on (1) liver transplantation prior to ICU admission; (2) ICU admission for the purposes of monitoring following elective surgery; (3) receipt of therapeutic anticoagulation prior to acquisition of TEG; or (4) receipt of FFP, platelets, or fibrinogen within 7 days prior to acquisition of TEG. With these exclusion criteria in place, our final cohort ultimately represented critically ill patients with cirrhosis who did not receive blood products or anticoagulation prior to TEG collection (Figure 1).

As depicted in Figure 1, of the 1996 patients with cirrhosis admitted to the ICU, 712 (36%) had TEG measured during their ICU stay. Of these, 336 patients were excluded due to having received a liver transplantation prior to TEG measurement. An additional 187 patients were excluded due to being admitted to the ICU only for the purpose of an elective surgery, mostly coronary artery bypass graft. Of the remaining 189 patients, 51 were excluded due to receipt of blood products prior to TEG measurement, and an additional 86 were excluded due to receipt of therapeutic anticoagulation at the time of TEG measurement.

The primary endpoint of this study was 28‐day mortality. Demographic data, etiology of liver disease, indication for hospitalization, and clinical/laboratory data necessary for calculation of MELD and characterization of ACLF were also extracted.

### 2.2. TEG

All TEG assays were collected as a part of routine clinical practice and not a part of a prospective research study. All TEG assays were performed within 72 h of either hospital admission or transfer to the ICU. For those patients who had multiple TEG assays performed during their hospitalization, the earliest result was used for analysis. TEG assays were performed on a standard Haemoscope TEG 5000 Thrombelastograph Hemostasis System [26]. Data for individual TEG parameters (R time, K time, alpha angle, MA) were extracted from the EMR for analysis, and a standard formula was used to calculate the Coagulation Index (a global assessment of coagulability) when relevant [27].

### 2.3. ACLF Definition

The ACLF‐CLIF Consortium definition was used to identify patients with ACLF [23]. Due to the retrospective nature of the study, we did not have access to West‐Haven scores to use for defining cerebral failure. Instead, cerebral failure was defined as a Glasgow Coma Scale (GCS) score < 12, which has previously been shown to correlate with West‐Haven Grade 3‐4 encephalopathy [28]. For those patients in the cohort who were mechanically ventilated (*n* = 17), GCS was assessed prior to intubation. Otherwise, we were able to access bilirubin levels, serum creatinine and/or need for renal replacement therapy, INR, mean arterial pressure and/or vasopressor requirements, and SpO_2_/FiO_2_ ratio. The specific definitions for organ failures (OFs) and ACLF grade used for our study are outlined in Table 1. For our modified TEG‐ACLF definition, we have replaced the INR with maximum amplitude (MA), as discussed in more detail later in the Results section of the text.

**TABLE 1:** To accommodate for the lack of access to the West‐Haven score in our retrospective cohort, we developed a modified scoring system for ACLF definition and grading, labeled modified EASL‐CLIF, which uses the Glasgow Coma Scale as a surrogate marker for cerebral failure.

**Organ failure**	**EASL-CLIF**	**Modified EASL-CLIF**	**TEG-ACLF**

Liver	Bilirubin > 12 mg/dL	Bilirubin > 12 mg/dL	Bilirubin > 12 mg/dL
Kidney	Creatinine > 2.0 or RRT	Creatinine > 2.0 or RRT	Creatinine > 2.0 or RRT
Brain	West‐Haven score 3‐4	Glasgow Coma score < 12	Glasgow Coma score < 12
Coagulation	INR > 2.5	INR > 2.5	MA < 55 mm
Circulatory	Requirement for vasopressor therapy	Requirement for vasopressor therapy	Requirement for vasopressor therapy
Respiratory	PaO_2_/FiO_2_ < 200 or SpO_2_/FiO_2_ < 214	PaO_2_/FiO_2_ < 200 or SpO_2_/FiO_2_ < 214	PaO_2_/FiO_2_ < 200 or SpO_2_/FiO_2_ < 214

**ACLF Grade**	**EASL-CLIF**	**Modified EASL-CLIF**	**TEG-ACLF**

Grade 1	1. Single kidney failure 2. Single nonkidney OF plus creatinine 1.5–1.9 mg/dL OR WH score 1‐2	1. Single kidney failure 2. Single nonkidney OF plus creatinine 1.5–1.9 mg/dL	1. Single kidney failure 2. Single nonkidney OF plus creatinine 1.5–1.9 mg/dL

Grade 2	Presence of 2 OF	Presence of 2 OF	Presence of 2 OF

Grade 3	Presence of 3 or more OF	Presence of 3 or more OF	Presence of 3 or more OF

*Note:* Later, we made an additional modification with the replacement of MA with INR in the definition of coagulation failure, labeled TEG‐ACLF. Entry‐level criteria for all definitions include cirrhosis with decompensation.

### 2.4. Statistical Analysis

Continuous variables are summarized as mean (standard deviation) and discrete variables as frequency (percentage). Differences in TEG parameters between subjects who did and did not survive beyond 28 days were compared using the Wilcoxon rank sum test. Logistic regression was used to estimate adjusted odds ratios for TEG parameters with 28‐day mortality. TEG parameters were scaled according to their IQR, so that the odds ratios may be interpreted as the expected effect if a subject’s measure was changed from the 25th to the 75th percentile. When adjusting for platelet count, the log of the platelet count was used. The best cutoffs were identified as those that maximized the average of the sensitivity and specificity. All hypothesis tests were two‐tailed with a 0.05 significance level. Given the exploratory nature of the analysis, no adjustments were made for multiple hypotheses. Analyses were conducted using R software, Version 4.2 (R Foundation for Statistical Computing, Vienna, Austria).

## 3. Results

As outlined in Figure 1, the final cohort included 52 critically ill patients with cirrhosis who had TEG collected within their ICU admission and did not receive therapeutic anticoagulation or blood products (RBCs, platelets, fibrinogen, FFP, or cryoprecipitate) prior to TEG collection. The cohort had a female predominance (58%) and an average age of 61 (range 34–90) (Table 2). Most patients had either alcohol‐associated liver disease or metabolic dysfunction–associated steatotic liver disease (MASLD) as the underlying cause of cirrhosis. In total, 33/52 (63%) met criteria for ACLF.

**TABLE 2:** Cohort characteristics.

Demographic information	
Male (%)	22 (42%)
Female (%)	30 (58%)
Average age (range)	61 (34–90)
Average BMI (range)	27 (16.7–40.5)
Underweight (%)	3 (6%)
Obese (%)	13 (25%)
Comorbid conditions	
Diabetes mellitus (%)	17 (33%)
Chronic kidney disease (%)	17 (33%)
Coronary artery disease (%)	12 (23%)
Pulmonary hypertension (%)	9 (17%)
Hypertension (%)	8 (15%)
COPD (%)	8 (15%)
Prior stroke (%)	5 (10%)
Etiology of advanced liver disease	
Alcohol‐related (%)	24 (46%)
MASLD (%)	14 (27%)
Viral hepatitis (%)	5 (10%)
Primary biliary cirrhosis (%)	4 (8%)
Primary sclerosing cholangitis (%)	2 (4%)
Other (%)	3 (6%)
Severity of liver disease	
Average MELD	24 (21–27)
Average INR (IU)	2.2 (1.7–2.5)
Average sodium (mEq/L)	137 (131–143)
Average creatinine (mg/dL)	1.8 (1.1–2.5)
Average bilirubin (mg/dL)	7.1 (5.1–9.3)
Average platelet count	123 (99–160)
Average WBC count	11.6 (9.4–13.1)
Categorization of acute liver disease	
With acute decompensation (%)	41 (79%)
Hepatic encephalopathy (%)	25 (48%)
Variceal bleeding (%)	5 (10%)
Ascites (%)	32 (60%)
Without acute‐on‐chronic liver failure (%)	19 (37%)
Acute‐on‐chronic liver failure (%)	33 (63%)
Grade 1 (%)	6 (18%)
Grade 2 (%)	10 (30%)
Grade 3 (%)	17 (52%)
Reason for ICU admission	
Sepsis/septic shock (%)	19 (37%)
Alcohol‐associated hepatitis (%)	9 (17%)
Gastrointestinal hemorrhage	7 (13%)
Bowel obstruction or volvulus (%)	7 (13%)
Intracranial hemorrhage (%)	5 (10%)
Other (%)	5 (10%)
Reason for death or dismissal to hospice	
ACLF following sepsis/septic shock (%)	7 (35%)
ACLF with GI bleeding trigger (%)	4 (20%)
ACLF following major abdominal surgery (%)	3 (15%)
ACLF with alcohol or uncertain trigger (%)	3 (15%)
Intracranial hemorrhage	3 (15%)

A manual review of the EHR was performed to determine the indications for obtaining TEG in each patient. In total, 32 patients had TEG performed to assist in guiding transfusion of blood products (22 for FFP/cryoprecipitate and 10 for platelet transfusions). Most frequently, clinicians used TEG to justify avoiding blood products despite elevated INR or low platelet count, though some clinicians utilized an entirely TEG‐guided transfusion protocol. For 9 patients, TEG was used to assess potentially reversible coagulation deficits prior to invasive procedures. For 4 patients, TEG was ordered in the setting of intracranial hemorrhage without obvious inciting trauma (a cause for intracranial hemorrhage was not specifically identified for any of these patients). There were 3 cases for which TEG was used to assess for early DIC in the setting of septic shock [29]. Finally, there were 4 cases for which no indication was described in the medical record.

### 3.1. TEG Parameters Associated With Short‐Term Mortality

Of the 52 patients in the cohort, 20 did not survive beyond 28 days. As depicted in Table 3, all TEG parameters were significantly different between those who survived versus those who did not. The observed differences generally reflected a more hypocoagulable state in those patients who did not survive beyond 28 days, with more prolonged R time, prolonged K time, decreased alpha angle, and decreased MA. When adjusted for INR, alpha angle and MA remained significantly associated with mortality (OR: 0.35, 95% CI: 0.14–0.86 and 0.28, 95% CI: 0.10–0.84, respectively), whereas both R time and K time had ORs > 1.50, which did not reach statistical significance (Table 4). When adjusted for platelet count, only MA was significantly associated with mortality (OR: 0.26, 95% CI: 0.08–0.92). When adjusted for both INR and platelet count concurrently, no associations reached statistical significance.

**TABLE 3:** Comparison of TEG parameters in survivors versus nonsurvivors.

**Entire cohort**	**Alive (*N* = 32)**	**Deceased (*N* = 20)**	**p** **value**

R time (min)	7.6 (4.2)	10.0 (4.2)	0.033
K time (min)	2.2 (1.5)	3.4 (2.2)	0.003
Alpha angle (degrees)	63.5 (10.4)	53.1 (12.5)	0.002
MA (mm)	60.2 (9.8)	49.2 (11.4)	0.001
INR	1.8 (0.9)	2.7 (1.6)	0.015
MELD	19.1 (7.1)	31.5 (8.7)	< 0.001

**ACLF cohort**	**Alive (*N* = 16)**	**Deceased (*N* = 17)**	**p** **value**

R time (min)	8.5 (4.7)	10.9 (3.9)	0.040
K time (min)	2.3 (1.8)	3.7 (2.3)	0.003
Alpha angle (degrees)	62.4 (12.2)	50.8 (11.9)	0.003
MA (mm	60.2 (8.1)	47.3 (11.2)	< 0.001
INR	2.0 (1.0)	2.9 (1.6)	0.047
MELD	22.2 (5.6)	33.3 (7.1)	< 0.001

*Note:* Hypocoagulable TEG parameters are associated with mortality. Values are expressed as mean (SD).

**TABLE 4:** Association between TEG parameters and mortality, adjusted for INR and platelets (PLTs).

**Entire cohort**	**Odds ratio** ^†^	**Confidence interval**	**p** **value**

*Adjusted for INR*			
R time	1.61	0.74–3.48	0.23
K time	1.58	0.96–2.61	0.07
Alpha angle	0.35	0.14–0.86	0.02
MA	0.28	0.10–0.84	0.02

*Adjusted for PLT*			
R time	1.68	0.79–3.55	0.17
K time	1.37	0.82–2.30	0.23
Alpha angle	0.39	0.15–1.03	0.06
MA	0.26	0.08–0.92	0.04

*Adjusted for INR and PLT*			
R time	1.47	0.65–3.30	0.35
K time	1.34	0.79–2.26	0.27
Alpha angle	0.45	0.17–1.15	0.10
MA	0.41	0.11–1.56	0.19

**ACLF cohort**	**Odds ratio** ^†^	**Confidence interval**	***p* value**

*Adjusted for INR*			
R time	1.81	0.70–4.65	0.22
K time	1.71	0.92–3.18	0.09
Alpha angle	0.31	0.11–0.89	0.030
MA	0.12	0.02–0.66	0.015

*Adjusted for PLT*			
R time	1.82	0.71–4.65	0.21
K time	1.51	0.78–2.93	0.23
Alpha angle	0.34	0.10–1.10	0.07
MA	0.12	0.02–0.72	0.02

*Adjusted for INR and PLT*			
R time	1.65	0.64–4.25	0.30
K time	1.55	0.80–2.99	0.19
Alpha angle	0.36	0.11–1.13	0.08
MA	0.17	0.03–1.10	0.06

^†^Odds ratio as per change from 25th to 75th percentile.

In total, 33 patients met the ACLF‐CLIF Consortium definition for ACLF. Of this subgroup, 16 patients survived beyond 28 days, whereas 17 did not. All TEG parameters were significantly different between those who survived versus those who did not (Table 3). When adjusted for INR, MA (OR: 0.12, 95% CI: 0.02–0.66) and alpha angle (OR: 0.31, 95% CI: 0.11–0.89) were significantly associated with mortality. Both R time and K time had large ORs > 1.70, which did not reach statistical significance (Table 4). When adjusted for platelet count, MA was again the only parameter significantly associated with mortality (OR: 0.12, 95% CI: 0.02–0.72). When adjusted for both INR and platelet count concurrently, no associations reached statistical significance in the ACLF cohort.

The overall cohort demonstrated a mean coagulation index of 0.8 (95% CI: −0.8–2.4), which would generally reflect a state of rebalanced hemostasis common to patients with cirrhosis. Those patients who met criteria for ACLF tended to have more hypocoagulable TEG parameters as compared to those who did not meet criteria for ACLF, with a coagulation index of 0.2 (95% CI: −1.0–1.4) versus 2.0 (95% CI: 1.5–2.8), respectively (*p* = 0.004).

### 3.2. Application of TEG in an Alternative Definition for ACLF

MA was chosen as a marker for coagulation failure as a replacement for INR, due to its association with 28‐day mortality, even after adjusting for INR or platelet count. Based on normal ranges reported by our laboratory, as well as precedent set by prior authors, we defined coagulation failure as an MA value of < 55 mm [21, 30, 31]. Accordingly, an alternative definition for ACLF (TEG‐ACLF) was applied to the patient cohort, wherein “coagulation failure” was defined by an MA value < 55 mm, rather than an INR value > 2.5. All other aspects of the definition remained unchanged, as outlined in Table 1.

**FIGURE 2:**
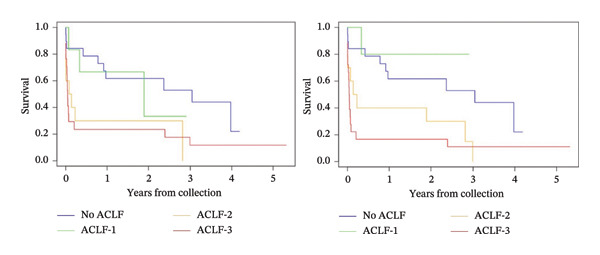
Kaplan–Meier (KM) curves for traditional ACLF (left) and TEG‐ACLF (right) grading systems.

**FIGURE 3:**
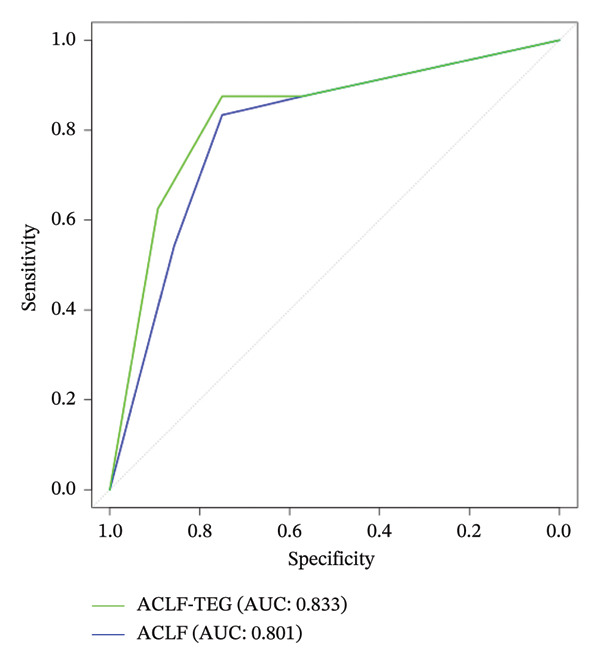
Receiver operating characteristic (ROC) curve for ACLF versus TEG‐ACLF.

In total, 5/52 (9.6%) patients were re‐classified into a different ACLF grade based on the alternative definition (TEG‐ACLF). Outcomes for these patients are detailed in Table 5. In the case of three patients, TEG‐ACLF grade was higher than traditional ACLF grade, and these patients suffered from short‐term mortality. For one patient, TEG‐ACLF grade was lower than traditional ACLF grade, and this patient survived for at least 2 years (after which follow‐up was lost). In these cases, TEG‐ACLF seemed to more accurately represent their risk of short‐term mortality than traditional ACLF. Kaplan‐Meier curves were also constructed for the cohort based on both the traditional ACLF definition and TEG‐ACLF (Figure 2). Overall, there was a trend toward a greater separation based on ACLF grade using the TEG‐ACLF definition, especially between grades 2 and 3. This is more clearly demonstrated by the changes in hazard ratios for each ACLF grade (Table 6). The HRs for traditional ACLF grades 2 and 3 were similar (2.8 and 2.9, respectively), whereas a greater difference for TEG‐ACLF was observed (2.58 and 3.4, respectively). A direct comparison is made between the two models via a receiver operating characteristic (ROC) curve, demonstrating a trend towards improved importance for prediction of short‐term mortality using TEG‐ACLF versus traditional ACLF, with an area under the curve (AUC) of 0.83 (0.72–0.95) versus 0.8 (0.68–0.92), respectively (Figure 3). This trend does not reach statistical significance (*p* = 0.12).

**TABLE 5:** Outcomes of those patients who were reclassified under the modified ACLF definition based on TEG.

	ACLF grade (traditional)	TEG‐ACLF grade	Outcome
PAtient 1	Grade 1	Grade 2	Living at last follow‐up (2 y)
PAtient 2	Grade 1	Grade 2	Deceased day 35
PAtient 3	Grade 2	Grade 3	Deceased day 23
Patient 4	Grade 2	Grade 3	Deceased day 3
Patient 5	Grade 3	Grade 2	Living at last follow‐up (2 y)

**TABLE 6:** Hazard ratios generated from KM curves for traditional ACLF and TEG ACLF grading systems.

ACLF grade:	Traditional ACLF (INR)		TEG‐ACLF (MA)	
	Hazard ratio (95% CI)	*p* value	Hazard ratio (95% CI)	*p* value
Grade 1	1.1 (0.3–4.2)	0.84	0.4 (0.1–3.3)	0.4
Grade 2	2.8 (1.1–7.2)	0.03	2.58 (1.0–6.4)	0.04
Grade 3	2.9 (1.3–6.6)	0.009	3.4 (1.5–7.7)	0.003

## 4. Discussion

Prediction of short‐term mortality in critically ill patients with cirrhosis is incredibly important for clinicians, patients, and families. In the past decade, ACLF has emerged as a defined clinical entity characterized by acute decompensation of cirrhosis, organ system failures, and substantial short‐term mortality [23, 32]. Apart from excellent supportive care and diligent investigation for reversible causes, there are no specific medical therapies with proven efficacy in the treatment of ACLF [33]. Because of this, rescue liver transplantation has been proposed as a cornerstone of therapy for this condition [34]. A variety of studies have demonstrated marked improvement in survival following liver transplantation, even among those patients with severe OFs on presentation [34, 35]. In fact, emerging evidence has suggested that early identification and expedited liver transplantation may lead to better outcomes, particularly in patients who present with ACLF Grade 3 [36, 37].

With transplantation in ACLF gaining additional support, it has become imperative to ensure that our definition of ACLF captures the complexity and mortality of patients as accurately as possible. As noted previously, “coagulation failure” as defined by INR > 2.5 is likely more representative of liver synthetic dysfunction rather than failure of coagulation [23]. Indeed, there is emerging evidence that assessment of coagulation by TEG provides prognostic information beyond the INR [20–22].

Our study adds to the growing body of literature supporting a prognostic role for TEG in the management of patients with cirrhosis and uniquely focuses on the prediction of short‐term mortality in the setting of critical illness. Although the size of our sample was relatively small, 28‐day mortality rates among the patients characterized as ACLF Grade 2 or Grade 3 were 50% and 70%, respectively, which closely align with rates reported in the literature and suggest that our small cohort is an appropriate representation of patients with ACLF [23, 32].

With respect to our primary outcome, more hypocoagulable TEG parameters were associated with increased 28‐day mortality. This aligns with available literature investigating the prognostic role of TEG in both the outpatient and inpatient settings [20–22, 38]. Interestingly, in our critically ill cohort, we demonstrated a stronger correlation between MA and mortality than had previously been described. This finding persisted even when corrected for platelet count, which suggests that the finding is at least partially driven by qualitative platelet dysfunction in those patients in our cohort at highest risk for mortality. Our small sample size may have inhibited our ability to detect a clinically significant association between R time and mortality; however, this had previously been suggested to be most strongly associated with ACLF [22]. Likewise, our small sample size likely explains the inability to detect an independent statistically significant association between any TEG parameters and mortality when adjusted for both platelet count and INR concurrently.

As proof of concept, we applied a modified TEG‐ACLF score (which redefined coagulation failure based on MA rather than INR) to our patient cohort. MA was chosen because it was the only parameter independently associated with mortality after adjusting for INR or platelet count, which are confounded by synthetic liver function and portal hypertension severity, respectively. This suggests that MA may serve as a more specific coagulation failure marker with a stronger impact on outcomes in our cohort. The most important observation from this exercise is that using MA rather than INR for defining coagulation failure appeared to more strongly predict mortality within our cohort, and it specifically generated a greater distinction between those patients with ACLF Grade 2 versus Grade 3. For two patients in the cohort, use of MA‐defined coagulation failure resulted in reclassification to ACLF Grade 3, and neither of these patients survived beyond 28 days. We hypothesize, therefore, that a modified ACLF grading system based on TEG may increase our sensitivity for detecting ACLF Grade 3, which we know represents a group of patients with dramatically higher mortality [23]. Especially considering that these patients may benefit from early liver transplantation, highly sensitive recognition of ACLF Grade 3 is critical [36, 37]. While a model such as TEG‐ACLF requires validation prior to being useful in clinical practice, our study provides a rationale for future examination of TEG as a tool for helping identify patients with high‐grade ACLF.

Since there are no standard indications for obtaining TEG in critically ill patients with cirrhosis, another strength of our study was the unique opportunity to investigate current practice patterns for applying TEG in this patient population. In our descriptive section, we found that the reported indication for ordering TEG and the subsequent response to abnormal TEG parameters were highly variable, highlighting the need for further research to inform clinicians on the best applications of this increasingly utilized diagnostic tool.

### 4.1. Limitations

The current study is not without limitations. Primarily, our sample size is small, owing largely to the retrospective study design, the elimination of patients who received blood products prior to TEG collection, and the relatively infrequent use of TEG in routine clinical practice. A larger cohort, for example, may have detected an independent association between mortality and other TEG parameters (specifically R time), which have been previously associated with mortality [21, 22]. In addition, due to missing data (LY30 was not reported in the majority of the cohort), we were unable to formally assess whether fibrinolysis was impaired in our patients. Impaired fibrinolysis has been previously linked to both hemorrhagic and thrombotic complications in cirrhosis, as well as poorer outcomes following liver transplantation, although its prognostic value outside of the context of liver transplantation is yet to be investigated [39, 40].

Since West‐Haven scores were not recorded in the medical record, we relied on an available surrogate marker of cerebral failure, the GCS score. Based on prior literature, a GCS score < 12 was chosen because it tends to correlate strongly with West‐Haven Grade 3‐4 encephalopathy [28]. Because of this, however, we had no way to reliably identify patients with milder (WH Grade 1‐2) encephalopathy, which does contribute to a portion of patients with Grade 1 ACLF as defined by CLIF‐EASL (Table 1) [23]. In the end, the impact of this was likely relatively small because most of the patients who had GCS < 12 in our cohort either had no evidence of hepatic decompensation (4/21) or already had > 3 noncerebral OFs (10/21).

Because acquisition of TEG was up to the discretion of the critical care physicians, the cohort presented here is highly selective and perhaps not adequately representative of the broad population of critically ill patients with cirrhosis. The information gained from TEG may have also had an impact on whether patients underwent invasive procedures, which could also have an impact on their mortality.

Finally, it cannot be ignored that while TEG is becoming more common, it remains a test for which access and expertise in interpretation are not universal. Certainly, more robust prospective studies are required to justify routine application to critically ill patients with cirrhosis in clinical practice.

## 5. Conclusions

In a cohort of critically ill patients with cirrhosis, we identified that hypocoagulable TEG parameters are associated with short‐term mortality. We also provide rationale for further prospective studies aimed at validating the association between TEG parameters and mortality, with the ultimate goal of incorporating TEG into existing prognostic tools to improve their accuracy.

**Nomenclature**
ACLF: Acute‐on‐chronic liver failure
CI: Confidence interval
EHR: Electronic health record
FiO_2_
: Fraction of inspired oxygen
FFP: Fresh frozen plasma
GCS: Glasgow Coma Scale
ICU: Intensive care unit
INR: International normalized ratio
MASLD: Metabolic dysfunction–associated steatohepatitis
MA: Maximum amplitude
OR: Odds ratio
R: Reaction time
RBCs: Red blood cells
SpO_2_
: Saturation of peripheral oxygen
TEG: Thromboelastography

## Author Contributions

Franklyn K. Wallace was responsible for primary data collection and manuscript preparation. Puru Rattan was involved in the initial data acquisition and refining the initial dataset. Ryan J. Lennon performed statistical analysis. Alice Gallo De Moraes and Douglas A. Simonetto offered mentorship, assistance with data interpretation, and editing of the final manuscript.

## Funding

The authors have nothing to report.

## Ethics Statement

Prior to collecting patient information, our study proposal was reviewed by our institutional review board (IRB) and granted an exemption based on its retrospective nature and deidentification of patient data (IRB number: 16‐002835). The study adhered to recognized ethical guidelines.

## Conflicts of Interest

The authors declare no conflicts of interest.

## Data Availability

Upon request, deidentified data may be made available to other investigators for incorporation in future studies.
